# Characterization of the PLN p.Arg14del Mutation in Human Induced Pluripotent Stem Cell-Derived Cardiomyocytes

**DOI:** 10.3390/ijms222413500

**Published:** 2021-12-16

**Authors:** Beatrice Badone, Carlotta Ronchi, Francesco Lodola, Anika E. Knaust, Arne Hansen, Thomas Eschenhagen, Antonio Zaza

**Affiliations:** 1 Laboratory of Cardiac Cellular Physiology, Department of Biotechnology and Bioscience, University of Milano-Bicocca, 20126 Milan, Italy; bba@sophion.com (B.B.); carlotta.ronchi@iit.it (C.R.); francesco.lodola@unimib.it (F.L.); 2 Department of Experimental Pharmacology and Toxicology, Cardiovascular Research Center, University Medical Center Hamburg-Eppendorf, 20246 Hamburg, Germany; a.knaust@uke.de (A.E.K.); ar.hansen@uke.de (A.H.); t.eschenhagen@uke.de (T.E.); 3 German Centre for Cardiovascular Research (DZHK), Partner Site Hamburg/Kiel/Lübeck, 20249 Hamburg, Germany

**Keywords:** phospholamban mutations, PLN p.Arg14del, PLN-SERCA2a interaction, hiPSC-CMs, dilated cardiomyopathy

## Abstract

Phospholamban (PLN) is the natural inhibitor of the sarco/endoplasmic reticulum Ca^2+^ ATP-ase (SERCA2a). Heterozygous PLN p.Arg14del mutation is associated with an arrhythmogenic dilated cardiomyopathy (DCM), whose pathogenesis has been attributed to SERCA2a “superinhibition”. Aim: To test in cardiomyocytes (hiPSC-CMs) derived from a PLN p.Arg14del carrier whether (1) Ca^2+^ dynamics and protein localization were compatible with SERCA2a superinhibition and (2) if functional abnormalities could be reverted by pharmacological SERCA2a activation (PST3093). Methods: Ca^2+^ transients (CaT) were recorded at 36 °C in hiPSC-CMs clusters during field stimulation. SERCA2a and PLN where immunolabeled in single hiPSC-CMs. Mutant preparations (MUT) were compared to isogenic wild-type ones (WT), obtained by mutation reversal. Results: WT and MUT differed for the following properties: (1) CaT time to peak (t_peak_) and half-time of CaT decay were shorter in MUT; (2) several CaT profiles were identified in WT, “hyperdynamic” ones largely prevailed in MUT; (3) whereas t_peak_ rate-dependently declined in WT, it was shorter and rate-independent in MUT; (4) diastolic Ca^2+^ rate-dependently accumulated in WT, but not in MUT. When applied to WT, PST3093 turned all the above properties to resemble those of MUT; when applied to MUT, PST3093 had a smaller or negligible effect. Preferential perinuclear SERCA2a-PLN localization was lost in MUT hiPSC-CMs. Conclusions: Functional data converge to argue for PLN p.Arg14del incompetence in inhibiting SERCA2a in the tested case, thus weakening the rationale for therapeutic SERCA2a activation. Mechanisms alternative to SERCA2a superinhibition should be considered in the pathogenesis of DCM, possibly including dysregulation of Ca^2+^-dependent transcription.

## 1. Introduction

The small sarcoplasmic reticulum (SR) transmembrane protein Phospholamban (PLN) is a crucial regulator of Ca^2+^ cycling in cardiomyocytes [1]. In the non-phosphorylated state, monomeric PLN partially inhibits the sarco/endoplasmic reticulum Ca^2+^-ATPase (SERCA2a), which operates Ca^2+^ reuptake from cytosol to the SR. Phosphorylation, by protein-kinase A (PKA), of PLN cytosolic domain (on Ser16) favors PLN pentamerization; this relieves the inhibition, thus increasing the rate of Ca^2+^ reuptake by SERCA2a under physiological conditions.

In the last decade, several PLN mutations have been found to associate with arrhythmogenic cardiomyopathy (ACM) and dilated cardiomyopathy (DCM) [2,3,4,5]. One of them, consisting of deletion of arginine at position 14 (PLN p.Arg14del), confers to DCM an increased risk of sudden cardiac death and need for heart transplantation [6]. This mutation, first discovered in a Greek family affected by DCM, has been classified as the most prevalent cardiomyopathy-related mutation in the Netherlands [5,6]. More than a decade ago, Haghighi and co-workers defined PLN p.Arg14del (in the clinically occurring heterozygous form) as “superinhibitory”, based on a larger than normal shift of SERCA2a Ca^2+^ sensitivity detected by in vitro measurements. The proposed mechanisms for superinhibition were an imbalance between the pentameric and monomeric PLN forms, resulting in an increase of the monomeric (inhibitory) one [7]. Further studies did not confirm a superinhibitory effect of PLN p.Arg14del in the basal state, but suggested that it might result from an impaired response of mutant PLN to PKA-mediated phosphorylation [8,9]. Although Ca^2+^ cycling abnormalities have been detected in cardiac myocytes carrying the mutation [10,11], it remains altogether unclear whether they indeed reflect SERCA2a superinhibition. An alternative to the “functional” interpretation of DCM pathogenesis has been provided by the presence of perinuclear PLN p.Arg14del aggregates, with potential toxicity, in myocardial biopsies of PLN p.Arg14del carriers [12] and in a transgenic murine model of the disease [13]. These additional findings suggest that mechanisms other than SERCA2a superinhibition may underlie the phenotype of this PLN mutation.

In this work we compare patient-specific PLN p.Arg14del induced pluripotent stem cell-derived cardiomyocytes (hiPSC-CMs) with their CRISPR-Cas9 isogenic controls, with the aim of detecting abnormalities in intracellular Ca^2+^ handling compatible with SERCA2a impairment. To validate our functional readouts of SERCA2a activity, we have compared mutation’s effects to those of (1) the SERCA2a inhibitor thapsigargin and (2) PST3093, a novel compound that enhances SERCA2a activity by inhibiting SERCA2a-PLN interaction [14]. This pharmacological investigation also aims to test the hypothesis that such SERCA2a activators may serve as a mechanism-targeted treatment of PLN p.Arg14del cardiomyopathy.

## 2. Results

### 2.1. Effect of PLN p.Arg14del on CaT Parameters and SR Ca^2+^ Content

**Figure 1:**
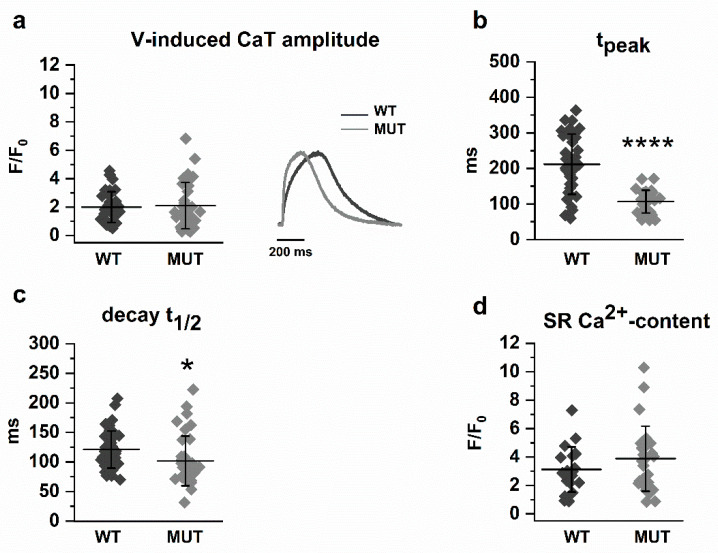
Parameters of V-induced CaT and SR Ca^2+^-content in WT and MUT. (**a**) CaT amplitude (WT *n* = 37, MUT *n* = 32); (**b**) CaT t_peak_ (WT *n* = 37, MUT *n* = 32); (**c**) CaT decay t_1/2_ (WT *n* = 43, MUT *n* = 36); (**d**) amplitude of caffeine-induced Ca^2+^ transients, reflective of SR Ca^2+^-content (WT *n* = 20, MUT *n* = 27); Data are expressed as mean ± SD; * *p* < 0.05 and **** *p* < 0.0001 vs. MUT.

Measurements were performed at steady-state (usually achieved within 10 beats) during stimulation at 1 Hz (Figure 1). CaT amplitude did not differ between MUT and WT (Figure 1a); however, CaT profiles differed between MUT and WT (see below), thus making the comparison between CaT amplitudes less meaningful. As part of CaT profile differences, both t_peak_ (Figure 1b) and the decay t_1/2_ (Figure 1c) were shorter in MUT than in WT, indicating faster SR Ca^2+^ uptake in the former. SR Ca^2+^-content had a large variance, with means similar between WT and MUT; if anything, cells with a high SR Ca^2+^ content were more represented in the MUT group (Figure 1d).

**Figure 2:**
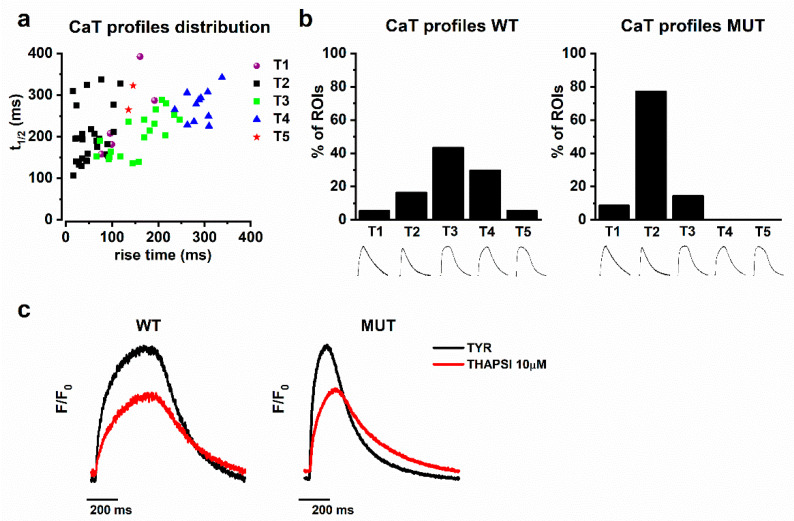
Distribution of CaT profiles and thapsigargin effect in WT and MUT. (**a**) CaT profiles from the WT and MUT groups are pooled and plotted according to their decay t_1/2_ vs. rise time. Data points are colored according to previous (blinded) profile assignement to a given type (T1, purple; T2, black; T3, green; T4, blue; T5, red). (**b**) Distribution of CaT profile types in WT (left) and MUT (right) preparations; profiles representative of each type are shown below the axis; (**c**) Representative WT (left) and MUT (right) CaT profiles in basal condition (TYR, black line) and after thapsigargin (THAPSI, red line).

As mentioned above, CaT profiles were heterogenous, but several patterns were identifiable. CaTs from all experimental groups were pooled (group identification blinded), and their profiles assigned to five visibly distinct categories (T1 to T5). By using the combination of their t_peak_ and decay t_1/2_, the CaT profiles thus identified formed distinct clusters (Figure 2). Albeit visibly distinct, T1 and T5 profiles were too rare to form a separate cluster. The T2 profile was characterized by fast rise and decay times, as commonly seen in mature myocytes (Figure 2a, black squares). The T4 profile combined a very slow rise with a slow decay (Figure 2a, blue triangles). The T3 profile was intermediate between T2 and T4 (Figure 2a, green circles). Notably, CaT amplitude was similar among all profile types.

While in WT the profiles were normally distributed (with median and mode in T3), in MUT there was a large prevalence of T2 (Figure 2b). Accordingly, T2 frequency was significantly higher in MUT (77.2% vs. 16.2%; *p* < 0.0001), whereas in WT T3 prevailed (43.2% vs. 14.3%, *p* < 0.01) and T4 was uniquely represented (29.7% vs. 0%; *p* < 0.05). Consistent with the different distribution of CaT profiles, t_peak_ and decay t_1/2_ were significantly shorter in MUT (Figure 1b,c).

We speculated that CaT profile differences between WT and MUT might reflect a different SERCA2a contribution to Ca^2+^ handling. To test this hypothesis, we exposed the preparations to thapsigargin (THAPSI, 10 µM), a SERCA2a inhibitor, and monitored CaT changes during steady-state pacing at 1 Hz. After 5 min of exposure, THAPSI changed CaT profile (from T2 to T4) in MUT only (Figure 2c). CaT amplitude was also progressively reduced, in both WT and MUT, to become nil at 20 min of THAPSI superfusion, thus confirming a crucial contribution of the SR store to Ca^2+^ handling in hiPSC-CMs.

Overall, opposite to what expected from depressed SERCA2a function, the PLN p.Arg14del mutation accelerated intracellular Ca^2+^ dynamics.

### 2.2. Effect of PST3093 on CaT Parameters and SR Ca^2+^-Content

**Figure 3:**
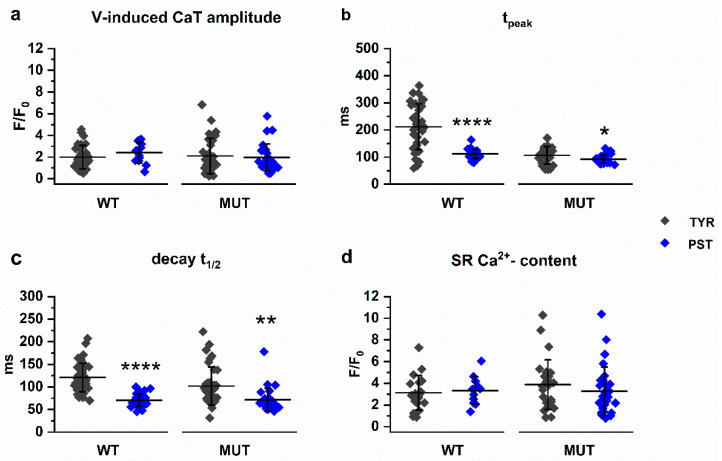
PST3093 1µM (PST) effect on V-induced CaT parameters and SR Ca^2+^-content in WT and MUT. (**a**) CaT amplitude in WT (TYR *n* = 37, PST *n* = 13) and MUT (TYR *n* = 32, PST *n* = 28); (**b**) t_peak_ in WT (TYR *n* = 37, PST *n* = 25) and MUT (TYR *n* = 32, PST *n* = 28); (**c**) Decay t_1/2_ in WT (TYR *n* = 43, PST *n* = 34) and MUT (TYR *n* = 36, PST *n* = 28); (**d**) SR Ca^2+^-content in WT (TYR *n* = 20, PST *n* = 12) and MUT (TYR *n* = 27, PST *n* = 28). Data are expressed as mean ± SD; * *p* < 0.05, ** *p* < 0.01, **** *p* < 0.0001 vs. TYR.

**Figure 4:**
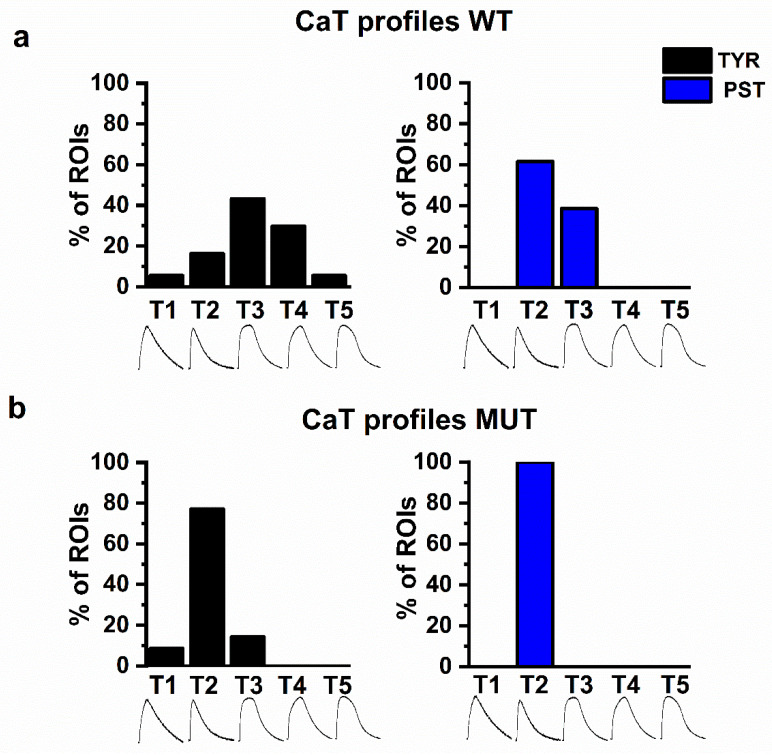
PST3093 1µM (PST) effect on the distribution of CaT profiles. Effect of PST on CaT profiles in WT (panel (**a**); TYR left, PST right) and MUT (panel (**b**); TYR left, PST right).

To test to which extent the consequences of the PLN p.Arg14del mutation reflected SERCA2a activation, we repeated the above experiments in WT and MUT preparations exposed to PST3093. PST3093 was tested at three different concentrations (100 nM, 500 nM, and 1 µM), in the range of those previously found to be effective in rat ventricular cardiomyocytes [2]. At concentrations up to 500 nM PST3093 effects did not achieve significance; therefore, only the effects of 1 µM PST3093 are reported here. Effects at all concentrations are described in the Supplementary Materials (Figures S1 and S2). PST3093 1 µM failed to affect CaT amplitude both in WT and MUT preparations (Figure 3a); nonetheless, it significantly changed the distribution of CaT profiles (Figure 4).

In WT preparations, PST3093 changed the normal distribution (containing all profiles) into one in which only T2 and T3 profiles were represented (Figure 4a). In PST3093-treated MUT preparations, T2 was the only CaT profile observed (Figure 4b). In both WT and MUT preparations, PST3093’s effect on profile distribution was statistically significant at 1 µM; nonetheless, a concentration-dependent trend was visible also at lower concentrations (Figure S2). PST3093 significantly shortened t_peak_ (Figure 3b) and decay t_1/2_ (Figure 3c) in WT preparations and, to a lesser extent (*p* < 0.05 vs. WT), in MUT ones. PST3093 failed to affect SR Ca^2+^ content in both WT and MUT preparations (Figure 3d).

To summarize, in WT preparations PST3093 “mimicked” the mutation and in MUT preparations PST3093 effect was smaller or negligible.

### 2.3. Effect of PLN p.Arg14del and PST3093 on Rate-Dependency of Ca^2+^ Dynamics

SERCA2a function affects the force-frequency relationship in cardiac myocytes [15,16]; therefore, we hypothesized that the PLN p.Arg14del mutation would affect the rate-dependency of intracellular Ca^2+^ dynamics in hiPSC-CMs.

**Figure 5:**
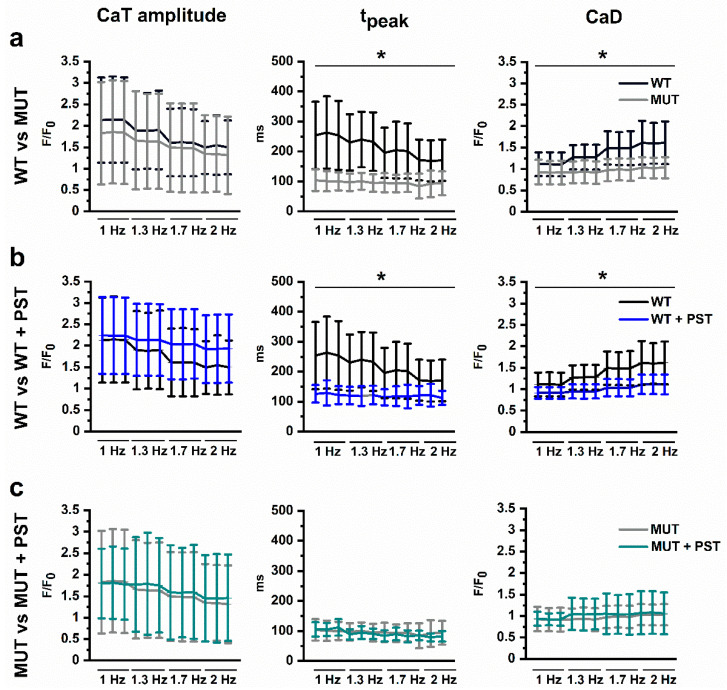
Rate-dependency of CaT parameters and CaD. Rate-dependency was tested by stepwise increments in pacing rate (1 Hz to 1.3 Hz, 1.7 Hz, and 2 Hz), the last 3 beats at steady-state are shown for each rate. (**a**) WT (black) vs. MUT (grey) in basal conditions (TYR; 9 ≤ *n* ≤ 15 for both groups); (**b**) PST3093 1µM (PST) effect in WT (TYR black, PST purple, 9 ≤ *n* ≤ 20); (**c**) PST effect in MUT (TYR grey, PST cyan; 9 ≤ *n* ≤ 20. Data are expressed as mean ± SD; * *p* < 0.05 for the interaction factor at two-way ANOVA.

We measured CaT features at four increasing stimulation frequencies (1, 1.3, 1.7, and 2 Hz) within the same ROI; the protocol was applied to compare WT and MUT preparations (Figure 5) and how they were affected by PST3093.

**Figure 6:**
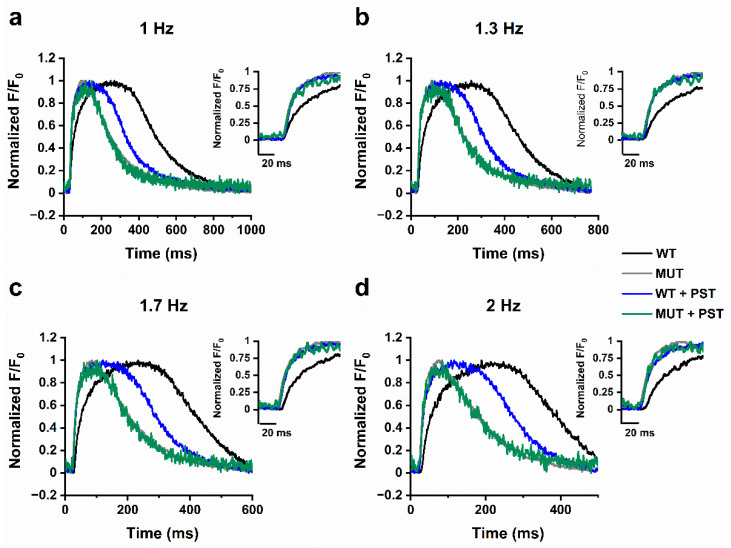
Rate-dependency of CaT profile in WT and MUT. Representative CaT profiles for WT and MUT in basal conditions and in presence of PST3093 1 µM (PST) at pacing rates of 1 Hz (**a**), 1.3 Hz (**b**), 1.7 Hz (**c**), and 2 Hz (**d**). The rising phase of CaT is shown at a faster time scale in the inset of each panel.

At variance with the pattern normally observed in mature human myocytes [17], both WT and MUT groups showed negative rate-dependency of CaT amplitude (i.e., smaller CaT amplitudes at higher rates). The steepness of such rate-dependency was similar between the two groups (Figure 5a, left). The most striking difference between WT and MUT was in the rate-dependency of t_peak_ (Figure 5a, middle). t_peak_ was longer in WT than in MUT at all rates (*p* < 0.05, two-way ANOVA); moreover, while it steeply decreased with rate in WT, it was short and almost insensitive to rate in MUT. As shown in the examples of Figure 6, in WT preparations T3-T4 profiles prevailed at low rate and were changed to approach T2 at higher rates. On the other hand, in MUT preparations the T2 profile type prevailed already at low rates and remained unchanged at higher ones (not shown). Diastolic Ca^2+^ (CaD) rate-dependency (Figure 5a, right), which mostly depends on the rate of cytosolic Ca^2+^ clearance, was steeply positive (implying cytosolic Ca^2+^ accumulation) in WT preparations and flat in MUT preparations (Figure 5a, right; *p* < 0.05, two-way ANOVA); this finding is consistent with the faster decay t_1/2_ observed in MUT preparations (see Figure 1b).

PST3093 tended to reduce the negative rate-dependency of CaT amplitude in WT; however, due to a large variance in the PST3093 group, the effect failed to achieve significance (Figure 5b, left). PST3093 did not affect rate-dependency of CaT amplitude in MUT preparations (Figure 5c, left). PST3093 significantly reduced rate-dependent CaD accumulation in WT preparations (Figure 5b, right, *p* < 0.01 at two-way ANOVA), but failed to affect the already flat rate-dependency of CaD in MUT ones (Figure 5c, right). The effect of PST3093 on CaD rate-dependency was present also at the lower drug concentrations (Figure S3).

PST3093 shortened t_peak_ and eliminated its rate-dependency in WT preparations (Figure 5b, middle and Figure 6, *p* < 0.01, two-way ANOVA), but it did not further modify t_peak_ or its rate-dependency in MUT ones (Figure 5c, middle and Figure 6). At all rates, PST3093 caused WT CaT profiles to become more similar to MUT ones (Figure 6).

To summarize, rate-dependency of CaD and t_peak_ sharply differed between WT and MUT preparations; PST3093 was ineffective in MUT ones and converted the WT pattern into one closely resembling the MUT.

### 2.4. Effect of PLN p.Arg14del on Electrical Activity

To evaluate the PLN p.Arg14del mutation effect on the electrical activity of hiPSC-CMs, spontaneous APs were recorded in WT and MUT hiPSC-CMs. The spontaneous beating rate was lower in MUT than in WT (40.6 ± 5.5 vs. 75.5 ± 9.7 b/min; *p* < 0.05; Figure S4a). Overall CL variability (SD_CL) was higher in MUT cells (Figure S4b). Illustrative Poincaré plots are shown in Figure S4d; the SD1/SD2 ratio did not differ between WT and MUT, to indicate an equal contribution of the short-term (beat-to beat) variability component in the two groups (Figure S4c).

C_APD50 and C_APD90 were slightly shorter in MUT than in WT cells (e.g., C_APD50 90.4 ± 13.0 vs. 113.7 ± 11.2 ms, *p* < 0.05; Figure S5a,b). In MUT APs, the plateau phase was less prominent and final repolarization was more gradual (see Figure S5f). No significant differences were detected between the two groups in maximal diastolic potential (MDP), AP amplitude (APA) and maximal upstroke velocity (dV/dt_max_) (Figure S5c–e).

A subset of preparations was stimulated at a constant rate (1 Hz) (Figure S6). Albeit APD50 and APD90 tended to be shorter in MUT than in WT (as the corresponding C_APD in spontaneous preparations), the differences did not achieve significance (Figure S6a,b). All the other AP parameters were similar between MUT and WT in paced preparations (Figure S6c–e).

Overall, the PLN p.Arg14del mutation had only minor effects on electrical activity.

### 2.5. PLN and SERCA2a Distribution and Colocalization

**Figure 7:**
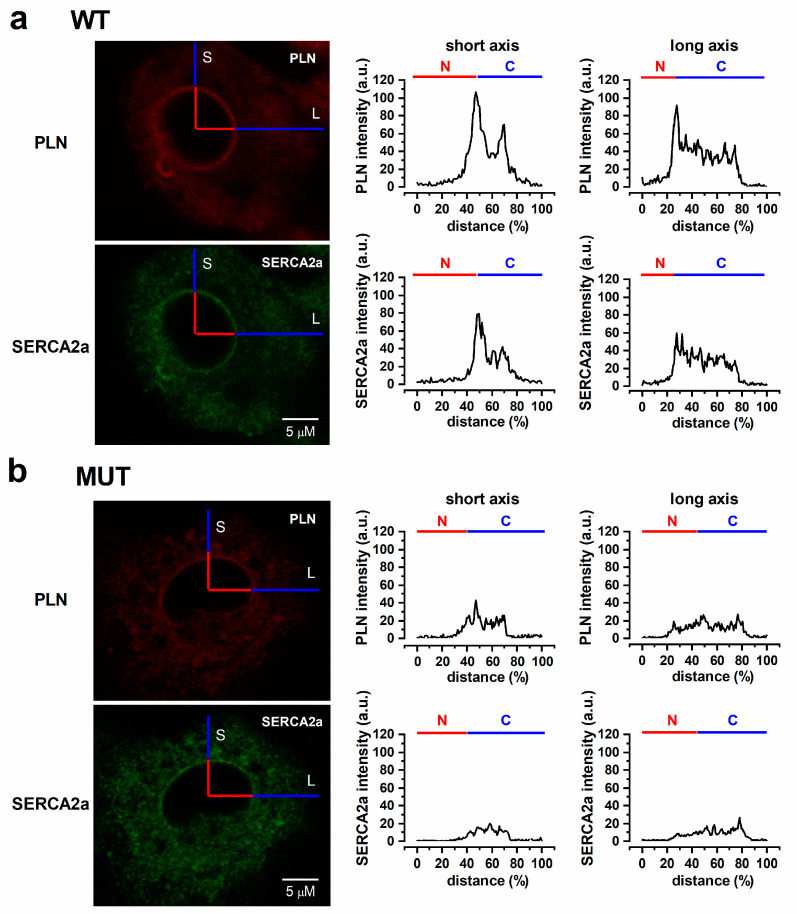
Subcellular PLN and SERCA2a localization in WT and MUT. Representative PLN and SERCA2a immunolabeling images (left) and the respective intensity profiles (distance from nucleus center expressed as % of total) for short and long cell axis (right) are shown. In the intensity profiles, the horizontal bars identify the perinuclear (N) and cytosolic (C) regions. (**a**) WT, (**b**) MUT. S = short axis, L = long axis.

**Figure 8:**
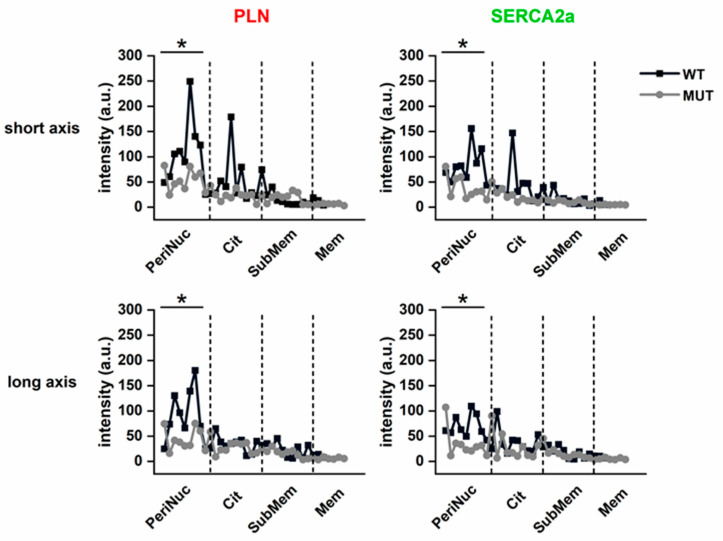
Average PLN and SERCA2a subcellular intensity profiles in WT and MUT. The PLN and SERCA2a intensity profiles (in the short and long cell axis) were binned and averaged among cells. WT (black, *n* = 9) and MUT (grey, *n* = 9). Subcellular regions are identified by the dotted lines: PeriNuc = perinuclear; Cyt = cytosolic; SubMem = subsarcolemma; Mem = sarcolemma. * *p* < 0.05 vs. MUT (by two-way ANOVA).

The differences in Ca^2+^ dynamics observed between MUT and WT preparations suggest that SERCA2a-PLN physical interaction might differ between the two groups. To test this hypothesis, intracellular distribution and colocalization of the two proteins were evaluated by immunostaining (Figure 7 and Figure 8).

SERCA2a (Ab green) and PLN (Ab red) were clearly detectable in both WT (Figure 7a) and MUT (Figure 7b) hiPSC-CMs as diffuse cytosolic fluorescence, with a substantial overlap between the two signals. To quantify subcellular distribution, pixel intensity profiles were obtained from orthogonal rasters, aligned with the long (L) and short (S) cell axis, respectively (Figure 7). Pearson’s correlation coefficient (PCC) was used to quantify the degree of colocalization between fluorophores (Figure S7).

In WT cells (*n* = 90, Figure 7a), both PLN and SERCA2a profiles peaked in the perinuclear region and, along the L axis, declined toward the sarcolemma; a smaller second peak was often present at the cytosolic level (at approx. 70% of raster) along the S axis. In MUT cells (*n* = 90; Figure 7b), the perinuclear signal for both PLN and SERCA was markedly blunted, but converged with that of WT cells when approaching the sarcolemma (along both cell axis). Statistics summarizing PLN and SERCA2a density profiles in MUT vs. WT cells are shown in Figure 8.

PLN-SERCA2a colocalization, quantified by the Pearsons’s correlation coefficient, was similar between the cytosol and the perinuclear regions and significantly, but only marginally, lower in MUT than in WT for both regions (Figure S7).

To summarize, the PLN p.Arg14del mutation only slightly decreased PLN/SERCA colocalization, but remarkably decreased the perinuclear density of both proteins.

## 3. Discussion

The functional aspects clearly discriminating MUT from WT were: (1) faster CaT rise (shorter t_peak_ and decay t_1/2_); (2) prevalence of type T2 CaT profiles, suggestive of a larger SERCA2a contribution; (3) absence of rate-dependent CaD accumulation and t_peak_ decrement; (5) shorter C_APD. PST3093 affected all the above Ca^2+^ dynamics parameters in WT only and it tended to convert the WT pattern into the MUT one.

The multiple functional differences between MUT and WT nicely converge into a pattern of accelerated systolic and diastolic Ca^2+^ dynamics suggestive of enhanced SR function. This view is reinforced by the conversion of the WT pattern into the MUT one by the SERCA2a activator.

In light of an enhanced SERCA2a function, the lack of differences in the amplitude of V-induced CaT (Figure 1) and its rate-dependency (Figure 5) might appear unexpected. Nonetheless, lack of CaT amplitude changes has been consistently reported under conditions of perturbed SERCA2a modulation by PLN, even if the latter was associated with significant mechanical derangement [18,19]. In particular, engineered heart tissue (EHT), also generated by our group from the same hiPSC-CMs used in the present study, was characterized by a marked decrease in force development in the face of normal CaT amplitude [20,21]. While the resilience of CaT amplitude to perturbations may be accounted for by a robust homeostatic control [22], deranged contraction in spite of unchanged Ca^2+^ signal would require additional explanations, such as a decrease in myofilament response to Ca^2+^, a hindrance to cell shortening or, as suggested by findings in EHTs [20], energetic incompetence. Since we did not measure contraction, this issue is not directly relevant to the present data; however, it should be considered when interpreting the pathophysiology of mutations leading to major contractile failure.

The constancy of the CaT amplitude contrasts with the presence of multiple CaT profiles, differentially represented in WT and MUT preparations (Figure 2). The prevalence of “slow” CaT profiles (T3, T4) in WT preparations might reflect the immaturity of the excitation–contraction (EC) coupling structure in hiPSC-CMs [23]. The observation that SERCA2a enhancement (by PST3093) changed the WT profile pattern to the MUT one (T2 prevalence, Figure 4) converges with the other results of the present study to suggest that the mutation may actually improve the SR Ca^2+^ uptake function. The mirror-image effect of SERCA2a inhibition (by thapsigargin, Figure 2c) reinforces this view. Notably, EHTs derived from the same WT hiPSC-CMs used in the present study did not display obvious heterogeneity of CaT profiles [20]. While probably reflecting different EC-coupling maturity, the heterogeneity in WT CaT profile serendipitously provided the present study with an additional readout of SERCA2a modulation by PLN p.Arg14del.

We anticipated that the effect of changes in SERCA2a function on CaT amplitude might be unveiled by its rate-dependency. In human ventricular myocytes, force (and CaT) rate-dependency is normally “positive” and its steepness decreases or reverses in contractile failure [17]. The present WT and MUT hiPSC-CM preparations were similarly characterized by “negative” rate-dependency of CaT amplitude instead (Figure 5), possibly because of the lack of appropriate “training” (mechanical and electrical) stimuli during maturation [24]. Why neither the PLN p.Arg14del mutation, nor PST3093, significantly changed the rate-dependency of CaT amplitude is unclear; nonetheless, the two conditions did affect the rate-dependency of the time to peak Ca^2+^ rise (t_peak_) and of diastolic Ca^2+^ (Figure 5). Changes in the latter parameters, again consistent between the mutation and PST3093 effects, are compatible with an increased dynamicity of Ca^2+^ handling.

Repolarization, particularly during the plateau phase, was somewhat faster in MUT than in WT (Figure S5). Whether this reflects a direct effect of the mutation on ion channels, or it is rather a consequence of perturbed Ca^2+^ handling, remains to be established. An example of the temporal relationship between membrane potential and Ca^2+^ transients in spontaneously beating WT and MUT cells (in which the two signals were simultaneously recorded) is shown in Figure S8.

Overall, the functional pattern we observed in MUT is clearly consistent with SERCA2a enhancement, i.e., a loss of PLN inhibitory function as a consequence of the mutation. This is in sharp contrast with the currently prevailing theory of a “superinhibitory” effect of heterozygous PLN p.Arg14del [7], which would imply a reduced SERCA2a function instead. The superinhibition theory comes largely from Ca^2+^ uptake assays by HEK-293 microsomes, in which PLN p.Arg14del overexpression markedly increased the K_m_ for Ca^2+^-dependent activation of transport [7]. Later work, using SERCA2a ATPase-activity as a readout, reported instead that PLN p.Arg14del was less effective than WT PLN in inhibiting SERCA2a [8,9]. Indeed, the mutation was found to stabilize a PLN conformation with a lower affinity for SERCA2a [9]. The same work reported that PLN p.Arg14del may be less sensitive to PKA-mediated phosphorylation [8,9], which implies loss of positive SERCA2a modulation by catecholamines. Notably, if PLN p.Arg14del inhibitory effects were small in the basal state, as our and others’ results suggest, adrenergic modulation of SERCA2a activity would be lost anyway, i.e., irrespective of PLN phosphorylation status. The PLN p.Arg14del mutation was reportedly associated with “irregularities” of Ca^2+^ transients during the spontaneous hiPSC-CMs beating and this could be reverted by correction of the mutation [10,11]. As the mechanism of such irregularities is unclear, this does not discriminate between gain and loss of function of mutant PLN.

Whereas SERCA2a superinhibition would have provided a suitable explanation for the clinical phenotype of PLN p.Arg14del carriers, i.e., contractile incompetence and electrical instability, loss of PLN inhibitory function is more difficult to reconcile with it. Permanently unhindered activity may increase ATP consumption by SERCA2a, ultimately leading to mitochondrial stress. However, Ca^2+^ recycling within the cell is energetically more efficient than its exchange with the extracellular space; thus, SERCA2a prevalence in the competition with the Na^+^/Ca^2+^ exchanger for cytosolic Ca^2+^ clearance should be globally energy-saving [25]. In terms of arrhythmogenesis, SERCA2a activation (e.g., by PLN knockout) may be protective, rather than proarrhythmic [26,27,28,29]. Altogether, an alternative hypothesis for the pathogenesis of the clinical phenotype should be considered. An intriguing one is that the effect of PLN p.Arg14del on myocardial function may be independent of PLN role in modulating EC coupling, as recently suggested by the observation that PLN R14del phenotype may be reverted by enhancement of the “unfolded protein response” [21].

Immunostaining of MUT hiPSC-CMs revealed a clear-cut reduction in the perinuclear PLN and SERCA2a signals (Figure 7 and Figure 8), as if the mutation impaired targeting of the protein complex to this compartment. On the other hand, the PLN-SERCA2a overlap was only slightly affected (Figure S7). Perinuclear SR is pivotal in Ca^2+^-dependent regulation of gene transcription, which adapts cell structure/function to the workload [30,31]. Defective Ca^2+^ accumulation in this compartment would likely disrupt this crucial adaptive response. The primarily “dilative” remodeling of PLN p.Arg14del-associated cardiomyopathy [6,13], which misses a proper “hypertrophic” phase, would be consistent with such a mechanism. Of course, based on the present results only, this is plain speculation; however, along with the major abnormalities in mitochondrial function detected in EHTs from the same hiPSC-CMs [20], it prompts to investigate Ca^2+^- or workload-dependent transcription in the PLN p.Arg14del mutation, an aim that should be ideally pursued in mechanically loaded preparations.

## 4. Limitations

While the evidence gathered in this study clearly converges to suggest that the mutation enhances SR function, this sharply conflicts with the concept of “superinhibition” reported by a pioneering study on the same mutation [7]. The hiPSC-CMs from which the present results were obtained originated from a single, albeit typical in terms of phenotype, mutation carrier. Thus, the present findings should not be generalized; they only indicate that a dysfunction other that SERCA2a superinhibition may underlie the dilated cardiomyopathy associated with the PLN p.Arg14del mutation. Whether this conclusion has a general value remains to be established.

## 5. Therapeutic Implications

The “superinhibition hypothesis” suggested that agents opposing PLN-SERCA2a interaction might target the very mechanism of the dilated cardiomyopathy associated with the mutation. Agents endowed with “PLN antagonism” have been identified and improved cardiac performance in patients with heart failure [32], a condition also characterized by overwhelming SERCA2a inhibition by PLN. The present results suggest that, at least in the case studied, the rationale for PLN antagonism as therapeutic approach would be much weaker, possibly limited to enhancement of the fraction of SERCA2a function still under the control of WT PLN. This is directly demonstrated by the consistent paucity of PST3093 effect on MUT preparations (Figure 4 and Figure 6), which is fully expected from a reduced PLN-SERCA2a interaction [33].

Whether PLN antagonism may affect SERCA2a targeting to the perinuclear compartment may deserve investigation.

## 6. Materials and Methods

### 6.1. hiPSC-CMs Preparation

PLN p.Arg14del hiPSC-CMs (MUT) were derived from a 31-year-old female patient carrying the mutation in the heterozygous form. The patient was affected by overt DCM with left ventricular dysfunction and ventricular arrhythmias. Control hiPSC-CMs were derived from the patient’s hiPSC in which the mutation was reversed by gene editing (CRISPR-Cas9 technology), thus allowing to test the mutation’s effect under otherwise isogenic conditions. No sequence changes beyond mutation reversal were detected, therefore control hiPSC-CMs are assumed to have a wild-type genotype and are hereafter abbreviated as WT. WT and MUT hiPSC-CMs were frozen at the 17th day post-differentiation. The methods for hiPSC preparation, cardiogenic differentiation and gene editing were recently published in detail [20].

### 6.2. Experimental Preparation

WT and MUT hiPSC-CMs vials were kept in liquid nitrogen until thawing, which was performed simultaneously within each batch. After thawing, cells were plated at a density of 200,000 cells/cm^2^ in 24-multiwell dishes coated with 1:100 Geltrex LDEV-Free matrix (Gibco by Life Technologies, Carlsbad, CA, USA; A14133). The culture medium contained DMEM low-glucose (Sigma Aldrich, St. Louis, MO, USA; D5921), 1% Penicillin/streptomycin (Sigma Aldrich, P0781), 10% Horse Serum (Gibco by Life Technologies, 26050-088), 10 µg/mL Human recombinant insulin (Sigma Aldrich, I9278), 33 µg/mL Aprotinin from bovine lung (Sigma Aldrich, A1153), 10 µmol/L RHO protein kinase inhibitor GSK 269962A (Biaffin GmbH & Co KG, Kassel, DE; 850664-21-0). The RHO protein kinase inhibitor was removed 24-h after thawing.

To reach a steady state condition, hiPSC-CMs were maintained for at least 2 weeks in the incubator (37 °C, 5% CO_2_). At days 28–30 post-differentiation, they were washed with room temperature PBS (Invitrogen, Waltham, MA, USA; 14040) and then detached with an enzyme cocktail (0.08 mL/cm^2^) consisting of StemPro Accutase (Gibco by Life Technologies, A11105) and Trypsin 2.5% (Gibco by Life Technologies, 15090), at room temperature from 20 up to 40 min. After seeding the suspension in dishes suitable for optical measurements (glass bottom) cells formed partially confluent beating monolayers (about 35,000 cells/1.5 cm^2^) on which measurements were performed.

### 6.3. Intracellular Ca^2+^ Recordings

Intracellular Ca^2+^ recordings were obtained between days 30 and 34 post differentiation. All experiments were performed at 36 °C under Tyrode’s (TYR) superfusion (adapted from ref [34]), consisting of (mM): 137 NaCl, 5.4 KCl, 2 CaCl_2_, 1 MgCl_2_, 10 Hepes, 10 Glucose, pH adjusted to 7.4 with NaOH. Ca^2+^ was optically measured in hiPSC-CMs incubated with Fluo-4AM (5 µM) for 20 min at 37 °C, and then washed for 5 min with TYR solution. All fluorescence (F) values were normalized to F_0_, which was recorded during prolonged quiescence. To collect local signals only (kinetics not distorted by propagation), a diaphragm in the optical path was adjusted to delimit a region of interest (ROI) corresponding to three/four cells. Four to five ROIs were analyzed in each plate. V-triggered Ca^2+^ transients (CaT) were measured during field stimulation. Baseline recordings were performed at 1 Hz; rate-dependency was tested by stepwise increments in pacing rate (to 1.3 Hz, 1.7 Hz, and 2 Hz). The following parameters were extracted from V-triggered CaT: diastolic Ca^2+^ (CaD), CaT amplitude (CaT peak—CaD), time to CaT peak (t_peak_) and half-time of CaT decay (decay t_1/2_). SR Ca^2+^-content was estimated from the amplitude of the Ca^2+^ surge elicited by an electronically timed caffeine (10 mM) pulse, applied for 5 s after steady-state stimulation at 1 Hz. To assess the contribution of the SR compartment to CaT profile, thapsigargin (10 µM) was applied for 5 min, a time sufficient to achieve partial SERCA2a blockade (steady-state thapsigargin superfusion abolished CaT almost completely).

### 6.4. Electrophysiological Recordings

Action potentials (AP) were recorded from spontaneously beating hiPSC-CMs by patch-clamp (I-clamp mode) in the whole-cell configuration at physiological temperature (36 °C). The uncompensated pipette resistance was in the range of 1.5–2.5 MΩ. The preparations were superfused with TYR (see above); the pipette (intracellular) solution contained (in mM): 110 K-aspartate, 23 KCl, 3 MgCl_2_, 0.04 CaCl_2_, 0.1 EGTA KOH (10^−7^ Ca^2+^-free), 5 Hepes KOH, 0.4 Na^+^-GTP, 5 Na^+^-ATP, 5 Na^+^-phosphocreatine, pH adjusted to 7.3 with KOH. APs were recorded either during spontaneous activity or during pacing. Beating clusters with ventricular-like APs were selected based on an APD90/APD50 ratio lower than 2.5 as suggested by APD values distribution in this cell population [35]. The following AP parameters were measured: firing rate (BPM), maximal diastolic potential (MDP), AP amplitude (APA); maximal upstroke velocity (dV/dt_max_); AP duration at 90% and 50% repolarization (APD90, APD50). During spontaneous activity, APD values were rate-corrected with Bazett’s formula, thus obtaining C_APD (C_APD90, C_APD50). To assess variability in beating rate the standard deviation (SD) of cycle lengths (CLs) was calculated as usual. To assess the relative weight of short-term (beat-to-beat) vs. long-term (over multiple cycles) CL variations, we graphed the CLs series in Poincarè plots (CL_n+1_ vs. CL_n_) and separately calculated the variability components orthogonal (SD1) and along (SD2) the identity line [36]. We have previously shown by numerical simulations that, whereas SD1 and SD2 covariates as a function of the total variance, the SD1/SD2 ratio reflects the frequency content of variance independent of total variance [37]. Thus, total variance (SD_CL) and the SD1/SD2 ratio are reported in the results; SD1/SD2 > 1 indicates the prevalence of the short-term component.

A subgroup of preparations was stimulated at 1 Hz (through the pipette) to compare AP parameters at a constant rate.

### 6.5. Immunofluorescence Staining

hiPSC-CMs plated in glass Petri dishes were rinsed twice in 1× PBS and fixed for 15 min with 4% paraformaldehyde at room temperature. Then, the preparations were washed twice with PBS, permeabilized and blocked in 2% FBS/2% BSA in PBS with 0.1% NP40 (blocking solution) for 45 min at room temperature. The preparations were incubated for 1 h in the blocking solution containing the primary antibodies: (a) SERCA2a C-20 (goat polyclonal, Santa Cruz) 1:100 and (b) PLN 2D12 (mouse monoclonal, Abcam) 1:50. The preparations were then washed three times in PBS for 5 min each. Secondary antibody incubation (45 min) consisted of (a) Alexafluor 488 anti-goat (donkey polyclonal, Life Technologies) 1:1500 for SERCA2a and (b) Alexafluor 647 anti-mouse (goat polyclonal, Life Technologies) 1:4000 for PLN, diluted in blocking solution. Preparations were washed three times in PBS and incubated in DAPI (1:1000) for 15 min to achieve nuclear staining. After a final two PBS washing steps, preparations were covered by PBS and stored at 4 °C in the dark. The preparations were imaged within 2 days by confocal microscopy; ImageJ was used to analyze the intensity and colocalization of PLN and SERCA2a signals. Cells were often elongated with the nucleus asymmetrically located; to rule out polarization, signals were quantified along the cell’s short and long axis separately.

### 6.6. Chemicals

We have identified and developed a compound endowed with SERCA2a stimulating effect, resulting from inhibition of PLN-SERCA2a interaction [33,38]. In order to induce functional SERCA2a upregulation, in this work, we used a derivative of this compound (PST3093), whose activity and selectivity for the target have been assessed by binding and functional studies [14]. We exposed preparations to PST3093 at three increasing concentrations (100 nM, 500 nM, and 1 µM) by incubation for 30 min at 37 °C.

PST3093 was dissolved in distilled water, thapsigargin in DMSO (final concentration < 0.1%). Except for PST3093, kindly provided by Windtree Therapeutics, all chemicals were purchased from Sigma Aldrich.

### 6.7. Statistics

OriginPro 2020 (OriginLab software, Northampton, MA, USA) and GraphPad Prism 8 (GraphPad software, San Diego, CA, USA) were used for statistical analysis. Pairs of means of continuous variables (amplitudes, t_1/2_) were compared by Student’s unpaired *t*-test. Categorical variables (frequency of CaT profiles) were compared by chi^2^-test. Two-way ANOVA was used to compare two treatment groups, each containing multiple means (parameters rate-dependency, immunofluorescence intensity). Pearson’s correlation coefficient (PCC, ImageJ) quantified PLN/SERCA2a colocalization in immunostaining experiments.

In most figures, individual data points are plotted, along with the sample mean ± SD, to illustrate dispersion. Statistical significance was defined as *p* < 0.05 *, *p* < 0.01 **, *p* < 0.001 *** and *p* < 0.0001 ****. Sample size (*n*) is specified for each experiment in the respective figure legend. For the sake of homogeneity of design with the comparison between WT and MUT preparations, PST3093’s effect was assessed by comparing plates incubated with PST3093 or its vehicle.

## Data Availability

The authors confirm that the data supporting the findings of this study are available within the article and/or its Supplementary Materials. The data that support the findings of this study are available from the corresponding author upon reasonable request.

## Supplementary Materials

The following are available online at https://www.mdpi.com/article/10.3390/ijms222413500/s1. References [39,40] are cited in the Supplementary Materials.

## References

[1] Kranias E.G., Hajjar R.J. (2012). Modulation of cardiac contractility by the phospholamban/SERCA2a regulatome. Circ. Res..

[2] Schmitt J.P., Kamisago M., Asahi M., Li G.H., Ahmad F., Mende U., Kranias E.G., MacLennan D.H., Seidman J.G., Seidman C.E. (2003). Dilated cardiomyopathy and heart failure caused by a mutation in phospholamban. Science.

[3] Ha K.N., Masterson L.R., Hou Z., Verardi R., Walsh N., Veglia G., Robia S.L. (2011). Lethal Arg9Cys phospholamban mutation hinders Ca^2+^-ATPase regulation and phosphorylation by protein kinase A. Proc. Natl. Acad. Sci. USA.

[4] Liu G.S., Morales A., Vafiadaki E., Lam C.K., Cai W.F., Haghighi K., Adly G., Hershberger R.E., Kranias E.G. (2015). A novel human R25C-phospholamban mutation is associated with super-inhibition of calcium cycling and ventricular arrhythmia. Cardiovasc. Res..

[5] Hof I.E., van der Heijden J.F., Kranias E.G., Sanoudou D., de Boer R.A., van Tintelen J.P., van der Zwaag P.A., Doevendans P.A. (2019). Prevalence and cardiac phenotype of patients with a phospholamban mutation. Neth. Heart J..

[6] Van der Zwaag P.A., van Rijsingen I.A., Asimaki A., Jongbloed J.D., van Veldhuisen D.J., Wiesfeld A.C., Cox M.G., van Lochem L.T., de Boer R.A., Hofstra R.M. (2012). Phospholamban R14del mutation in patients diagnosed with dilated cardiomyopathy or arrhythmogenic right ventricular cardiomyopathy: Evidence supporting the concept of arrhythmogenic cardiomyopathy. Eur. J. Heart Fail..

[7] Haghighi K., Kolokathis F., Gramolini A.O., Waggoner J.R., Pater L., Lynch R.A., Fan G.C., Tsiapras D., Parekh R.R., Dorn G.W. (2006). A mutation in the human phospholamban gene, deleting arginine 14, results in lethal, hereditary cardiomyopathy. Proc. Natl. Acad. Sci. USA.

[8] Ceholski D.K., Trieber C.A., Young H.S. (2012). Hydrophobic imbalance in the cytoplasmic domain of phospholamban is a determinant for lethal dilated cardiomyopathy. J. Biol. Chem..

[9] Vostrikov V.V., Soller K.J., Ha K.N., Gopinath T., Veglia G. (2015). Effects of naturally occurring arginine 14 deletion on phospholamban conformational dynamics and membrane interactions. Biochim. Biophys. Acta.

[10] Karakikes I., Stillitano F., Nonnenmacher M., Tzimas C., Sanoudou D., Termglinchan V., Kong C.W., Rushing S., Hansen J., Ceholski D. (2015). Correction of human phospholamban R14del mutation associated with cardiomyopathy using targeted nucleases and combination therapy. Nat. Commun..

[11] Stillitano F., Turnbull I.C., Karakikes I., Nonnenmacher M., Backeris P., Hulot J.S., Kranias E.G., Hajjar R.J., Costa K.D. (2016). Genomic correction of familial cardiomyopathy in human engineered cardiac tissues. Eur. Heart J..

[12] Te Rijdt W.P., van Tintelen J.P., Vink A., van der Wal A.C., de Boer R.A., van den Berg M.P., Suurmeijer A.J. (2016). Phospholamban p.Arg14del cardiomyopathy is characterized by phospholamban aggregates, aggresomes, and autophagic degradation. Histopathology.

[13] Eijgenraam T.R., Boukens B.J., Boogerd C.J., Schouten E.M., van de Kolk C.W.A., Stege N.M., Te Rijdt W.P., Hoorntje E.T., van der Zwaag P.A., van Rooij E. (2020). The phospholamban p.(Arg14del) pathogenic variant leads to cardiomyopathy with heart failure and is unreponsive to standard heart failure therapy. Sci. Rep..

[14] Arici M., Ferrandi M., Hsu S.-C., Torre E., Barassi P., Luraghi A., Ronchi C., Chang G.-J., Peri F., Ferrari P. (2021). Istaroxime metabolite PST3093 selectively stimulates SERCA2a and reverses disease-induced changes in cardiac function. bioRxiv.

[15] Huke S., Liu L.H., Biniakiewicz D., Abraham W.T., Periasamy M. (2003). Altered force-frequency response in non-failing hearts with decreased SERCA pump-level. Cardiovasc. Res..

[16] Balcazar D., Regge V., Santalla M., Meyer H., Paululat A., Mattiazzi A., Ferrero P. (2018). SERCA is critical to control the Bowditch effect in the heart. Sci. Rep..

[17] Pieske B., Maier L.S., Bers D.M., Hasenfuss G. (1999). Ca^2+^ handling and sarcoplasmic reticulum Ca^2+^ content in isolated failing and nonfailing human myocardium. Circ. Res..

[18] Zhai J., Schmidt A.G., Hoit B.D., Kimura Y., MacLennan D.H., Kranias E.G. (2000). Cardiac-specific overexpression of a superinhibitory pentameric phospholamban mutant enhances inhibition of cardiac function in vivo. J. Biol. Chem..

[19] Haghighi K., Schmidt A.G., Hoit B.D., Brittsan A.G., Yatani A., Lester J.W., Zhai J., Kimura Y., Dorn G.W., MacLennan D.H. (2001). Superinhibition of sarcoplasmic reticulum function by phospholamban induces cardiac contractile failure. J. Biol. Chem..

[20] Cuello F., Knaust A.E., Saleem U., Loos M., Raabe J., Mosqueira D., Laufer S., Schweizer M., van der Kraak P., Flenner F. (2021). Impairment of the ER/mitochondria compartment in human cardiomyocytes with PLN p.Arg14del mutation. EMBO Mol. Med..

[21] Feyen D.A.M., Perea-Gil I., Maas R.G.C., Harakalova M., Gavidia A.A., Arthur Ataam J., Wu T.H., Vink A., Pei J., Vadgama N. (2021). Unfolded Protein Response as a Compensatory Mechanism and Potential Therapeutic Target in PLN R14del Cardiomyopathy. Circulation.

[22] Venetucci L.A., Trafford A.W., O’Neill S.C., Eisner D.A. (2008). The sarcoplasmic reticulum and arrhythmogenic calcium release. Cardiovasc. Res..

[23] Parikh S.S., Blackwell D.J., Gomez-Hurtado N., Frisk M., Wang L., Kim K., Dahl C.P., Fiane A., Tonnessen T., Kryshtal D.O. (2017). Thyroid and Glucocorticoid Hormones Promote Functional T-Tubule Development in Human-Induced Pluripotent Stem Cell-Derived Cardiomyocytes. Circ. Res..

[24] Ronaldson-Bouchard K., Ma S.P., Yeager K., Chen T., Song L., Sirabella D., Morikawa K., Teles D., Yazawa M., Vunjak-Novakovic G. (2018). Advanced maturation of human cardiac tissue grown from pluripotent stem cells. Nature.

[25] Sakata S., Lebeche D., Sakata N., Sakata Y., Chemaly E.R., Liang L.F., Tsuji T., Takewa Y., del Monte F., Peluso R. (2007). Restoration of mechanical and energetic function in failing aortic-banded rat hearts by gene transfer of calcium cycling proteins. J. Mol. Cell. Cardiol..

[26] Davia K., Bernobich E., Ranu H.K., del Monte F., Terracciano C.M., MacLeod K.T., Adamson D.L., Chaudhri B., Hajjar R.J., Harding S.E. (2001). SERCA2A overexpression decreases the incidence of aftercontractions in adult rabbit ventricular myocytes. J. Mol. Cell. Cardiol..

[27] Del Monte F., Lebeche D., Guerrero J.L., Tsuji T., Doye A.A., Gwathmey J.K., Hajjar R.J. (2004). Abrogation of ventricular arrhythmias in a model of ischemia and reperfusion by targeting myocardial calcium cycling. Proc. Natl. Acad. Sci. USA.

[28] Bai Y., Jones P.P., Guo J., Zhong X., Clark R.B., Zhou Q., Wang R., Vallmitjana A., Benitez R., Hove-Madsen L. (2013). Phospholamban knockout breaks arrhythmogenic Ca^2+^ waves and suppresses catecholaminergic polymorphic ventricular tachycardia in mice. Circ. Res..

[29] Zaza A., Rocchetti M. (2015). Calcium store stability as an antiarrhythmic endpoint. Curr. Pharm. Des..

[30] Wu X., Zhang T., Bossuyt J., Li X., McKinsey T.A., Dedman J.R., Olson E.N., Chen J., Brown J.H., Bers D.M. (2006). Local InsP3-dependent perinuclear Ca^2+^ signaling in cardiac myocyte excitation-transcription coupling. J. Clin. Investig..

[31] Guo A., Cala S.E., Song L.S. (2012). Calsequestrin accumulation in rough endoplasmic reticulum promotes perinuclear Ca^2+^ release. J. Biol. Chem..

[32] Carubelli V., Zhang Y., Metra M., Lombardi C., Felker G.M., Filippatos G., O’Connor C.M., Teerlink J.R., Simmons P., Segal R. (2020). Treatment with 24 h istaroxime infusion in patients hospitalised for acute heart failure: A randomised, placebo-controlled trial. Eur. J. Heart Fail..

[33] Ferrandi M., Barassi P., Tadini-Buoninsegni F., Bartolommei G., Molinari I., Tripodi M.G., Reina C., Moncelli M.R., Bianchi G., Ferrari P. (2013). Istaroxime stimulates SERCA2a and accelerates calcium cycling in heart failure by relieving phospholamban inhibition. Br. J. Pharmacol..

[34] Zhang X.H., Haviland S., Wei H., Saric T., Fatima A., Hescheler J., Cleemann L., Morad M. (2013). Ca^2+^ signaling in human induced pluripotent stem cell-derived cardiomyocytes (iPS-CM) from normal and catecholaminergic polymorphic ventricular tachycardia (CPVT)-afflicted subjects. Cell Calcium.

[35] Ronchi C., Bernardi J., Mura M., Stefanello M., Badone B., Rocchetti M., Crotti L., Brink P., Schwartz P.J., Gnecchi M. (2020). NOS1AP polymorphisms reduce NOS1 activity and interact with prolonged repolarization in arrhythmogenesis. Cardiovasc. Res..

[36] Brennan M., Palaniswami M., Kamen P. (2001). Do existing measures of Poincare plot geometry reflect nonlinear features of heart rate variability?. IEEE Trans. Biomed. Eng..

[37] Altomare C., Bartolucci C., Sala L., Bernardi J., Mostacciuolo G., Rocchetti M., Severi S., Zaza A. (2015). IKr Impact on Repolarization and Its Variability Assessed by Dynamic Clamp. Circ. Arrhythm. Electrophysiol..

[38] Rocchetti M., Besana A., Mostacciuolo G., Micheletti R., Ferrari P., Sarkozi S., Szegedi C., Jona I., Zaza A. (2005). Modulation of sarcoplasmic reticulum function by Na^+^/K^+^ pump inhibitors with different toxicity: Digoxin and PST2744 [(*E*,*Z*)-3-((2-aminoethoxy)imino)androstane-6,17-dione hydrochloride]. J. Pharmacol. Exp. Ther..

[39] Breckwoldt K., Letuffe-Breniere D., Mannhardt I., Schulze T., Ulmer B., Werner T., Benzin A., Klampe B., Reinsch M.C., Laufer S. (2017). Differentiation of cardiomyocytes and generation of human engineered heart tissue. Nat. Protoc..

[40] Frank S., Zhang M., Scholer H.R., Greber B. (2012). Small molecule-assisted, line-independent maintenance of human pluripotent stem cells in defined conditions. PLoS ONE.

