# Supplementary material for: Characterization of the PLN p.Arg14del Mutation in Human Induced Pluripotent Stem Cell-Derived Cardiomyocytes
Source: Int J Mol Sci. 2021 Dec 16;22(24):13500. doi: 10.3390/ijms222413500 (PMC8709382; doi:10.3390/ijms222413500)
Supplement: Supplementary file 1 [file ijms-22-13500-s001.zip › ijms-1487986-supplementary.pdf]

SUPPLEMENT TO:

Characterization of the PLN p.Arg14del mutation in human induced pluripotent stem cell-derived cardiomyocytes

Beatrice Badone [1], Carlotta Ronchi [1], Francesco Lodola [1], Anika E. Knaust [2,3], Arne Hansen [2,3], Thomas Eschenhagen [2,3] and Antonio Zaza [1,*]

[1] *Laboratory of Cardiac Cellular Physiology, Department of Biotechnology and Bioscience, University of Milano-Bicocca, 20126, Milan, Italy; bba@sophion.com (B.B.); carlotta.ronchi@iit.it (C.R.); francesco.lodola@unimib.it (F.L.)*
[2] *Department of Experimental Pharmacology and Toxicology, Cardiovascular Research Center, University Medical Center Hamburg-Eppendorf, 20246, Hamburg, Germany; a.knaust@uke.de (A.E.K.); ar.hansen@uke.de (A.H.); t.eschenhagen@uke.de (T.E.)*
[3] *German Centre for Cardiovascular Research (DZHK), partner site Hamburg/Kiel/Lübeck, 20249, Hamburg, Germany.*

SUPPLEMENTARY METHODS

Cardiogenic differentiation and gene editing.

WT and MUT hiPCS lines were differentiated into cardiomyocytes as previously described [39]. In brief, undifferentiated hiPSCs were expanded in FTDA media [40]. Embryoid bodies (EBs) were generated from dissociated hiPSCs in spinner flasks format for 24 hours. EBs were transferred to pluronic-coated T175 cell culture flasks. Mesoderm progenitors and cardiomyocytes were differentiated by growth factor/small molecule cocktails. Differentiated hiPSC-CM were dissociated with collagenase II (200 units/mL; Worthington, Lakewood, NJ, USA; LS004176) and aliquots were frozen in liquid nitrogen.

SUPPLEMENTARY RESULTS

Dose-dependency of PST3093 effect on CaT parameters and SR Ca^{2+}-content (Figure S1)

CaT amplitude was unmodified by PST3093 at all concentrations both in WT and in MUT (Figure S1a). Decay $t_{1/2}$, showed instead a dose-dependency of PST3093 effect in both WT and MUT, with a significant effect achieved at 1 μM (Figure S1b). PST3093 did not significantly affect caffeine-induced CaT amplitude (representative of SR Ca^{2+} content) at all concentrations in both WT and MUT (Figure S1c). At variance with the effect observed with PST3093 1 μM (see manuscript figure 4), PST3093 100 and 500 nM failed to change the distribution of CaT profiles in both WT and MUT (Figure S2). This finding is consistent with the PST3093 concentration required to shorten decay $t_{1/2}$ significantly (see above).

Effect of PST3093 500 nM on rate-dependency of Ca^{2+} dynamics (Figure S3)

Similar to PST3093 1 μM (see manuscript figure 5), PST3093 500 nM failed to affect the rate-dependency of CaT amplitude significantly. In MUT preparations only, PST3093 decreased CaT amplitude at all rates (Figure S3b, left); however, as shown at 1 Hz in figure S1, PST3093 effect on CaT amplitude lacked a consistent dose-dependency. At variance with PST3093 1 μM (see manuscript figure 5), PST3093 500 nM failed to affect rate-dependent CaD accumulation significantly in both WT and MUT (Figure S3 right).

SUPPLEMENTARY FIGURES

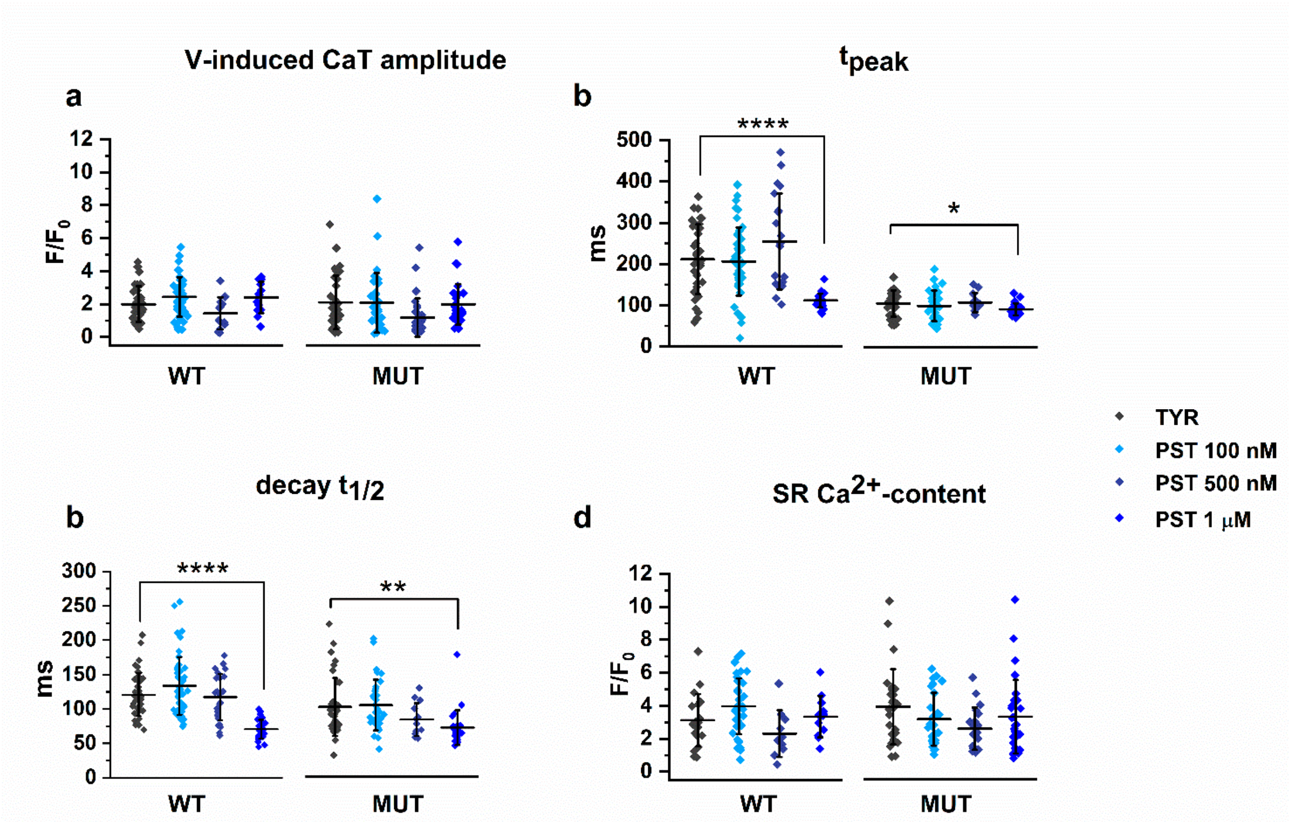

Figure S1. Dose-dependency of PST3093 (PST) effect on CaT parameters and SR Ca^{2+}-content in WT and MUT. In each dot plot PST 100, 500 nM, and 1 µM is compared to baseline (TYR) WT and MUT (n≥20 at all concentrations) **(a)** CaT amplitude **(b)** decay $t_{1/2}$; **(c)** SR Ca^{2+}-content; **(d)** Representative CaT profiles and their modulation by PST in WT (left) and MUT (right); **(e)** Representative caffeine-induced Ca^{2+} transients and their modulation by PST in WT and MUT (n≥20 at all concentrations). Data are expressed as mean ± SD; * $p < 0.05$, ** $p < 0.01$, **** $p < 0.0001$ vs TYR (ANOVA post-hoc).

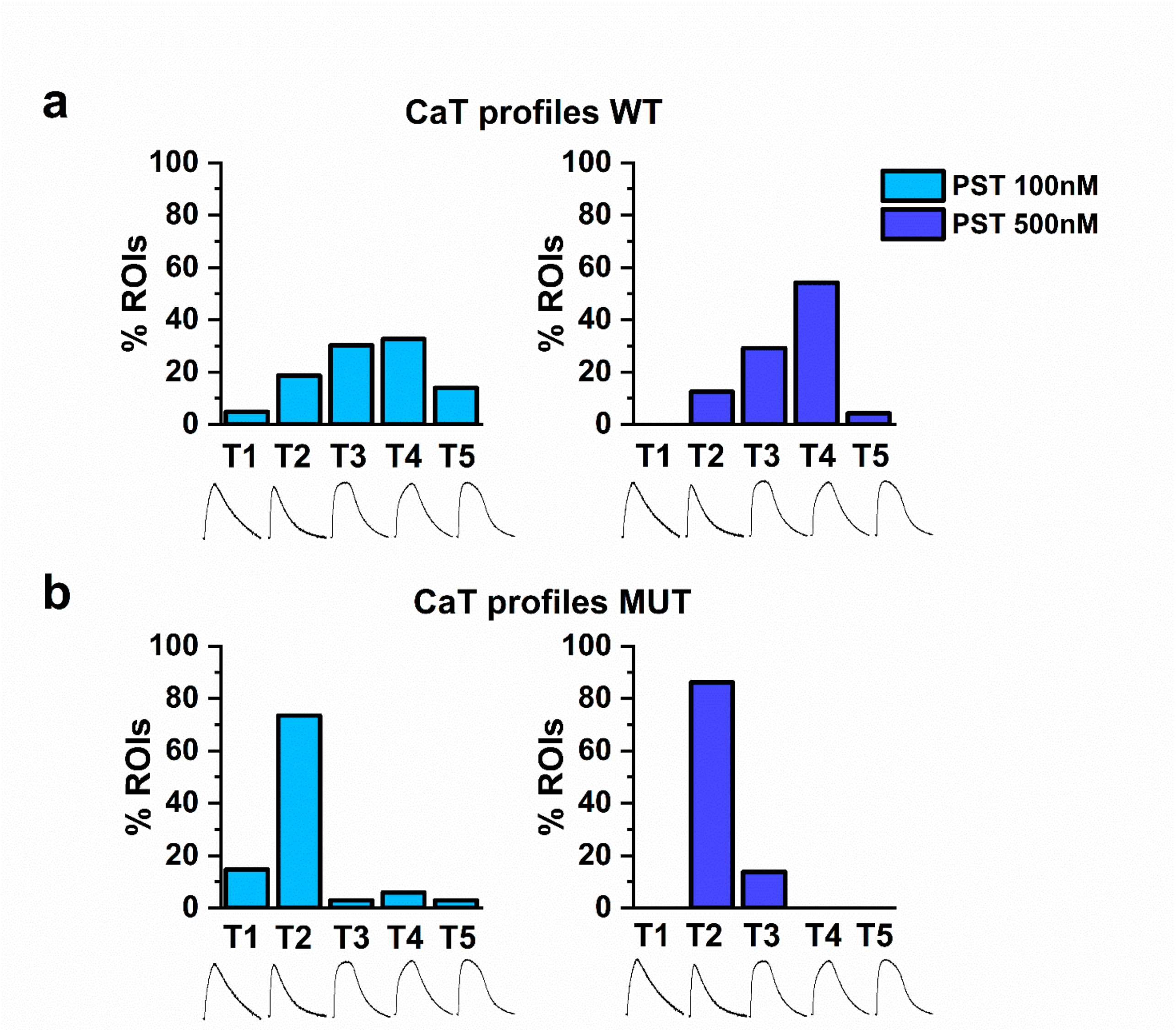

Figure S2. Dose-dependency of PST3093 (PST) effect on the distribution of CaT profiles in WT and MUT. Effect of PST 100, 500 nM, and 1 μM on CaT profiles; TYR is control. **(a)** WT; **(b)** MUT. Data for TYR and PST 1μM are those of manuscript Figure4 and are shown here for comparison.

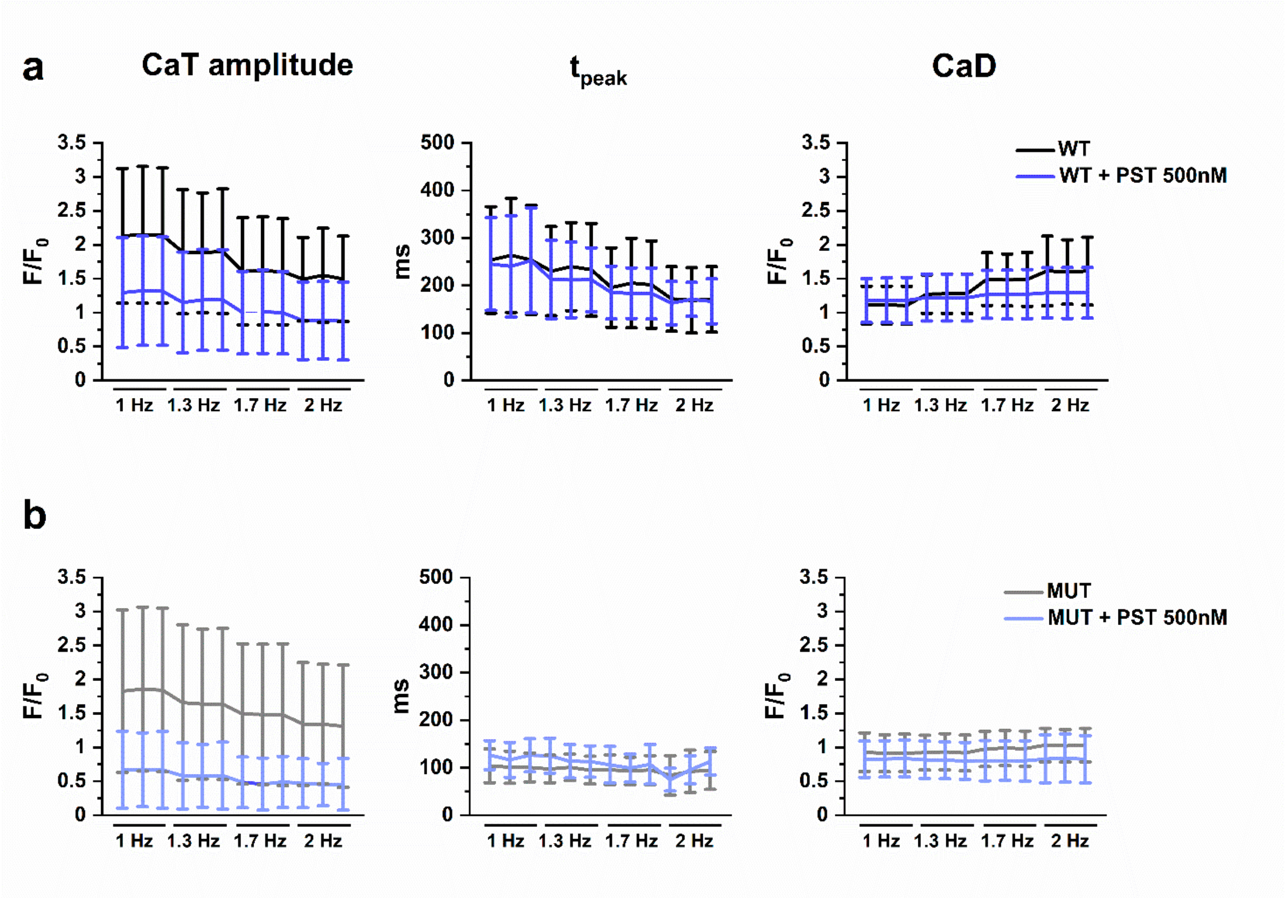

Figure S3. Effect of PST3093 (PST) 500 nM on the rate-dependency of CaT and CaD in WT and MUT. CaT amplitude (left panels), t_{peak} (middle panels) and CaD (right panels). **(a)** WT ($18 \le n \le 21$; NS); **(b)** MUT ($8 \le n \le 11$; NS). Data are expressed as mean ± SD.

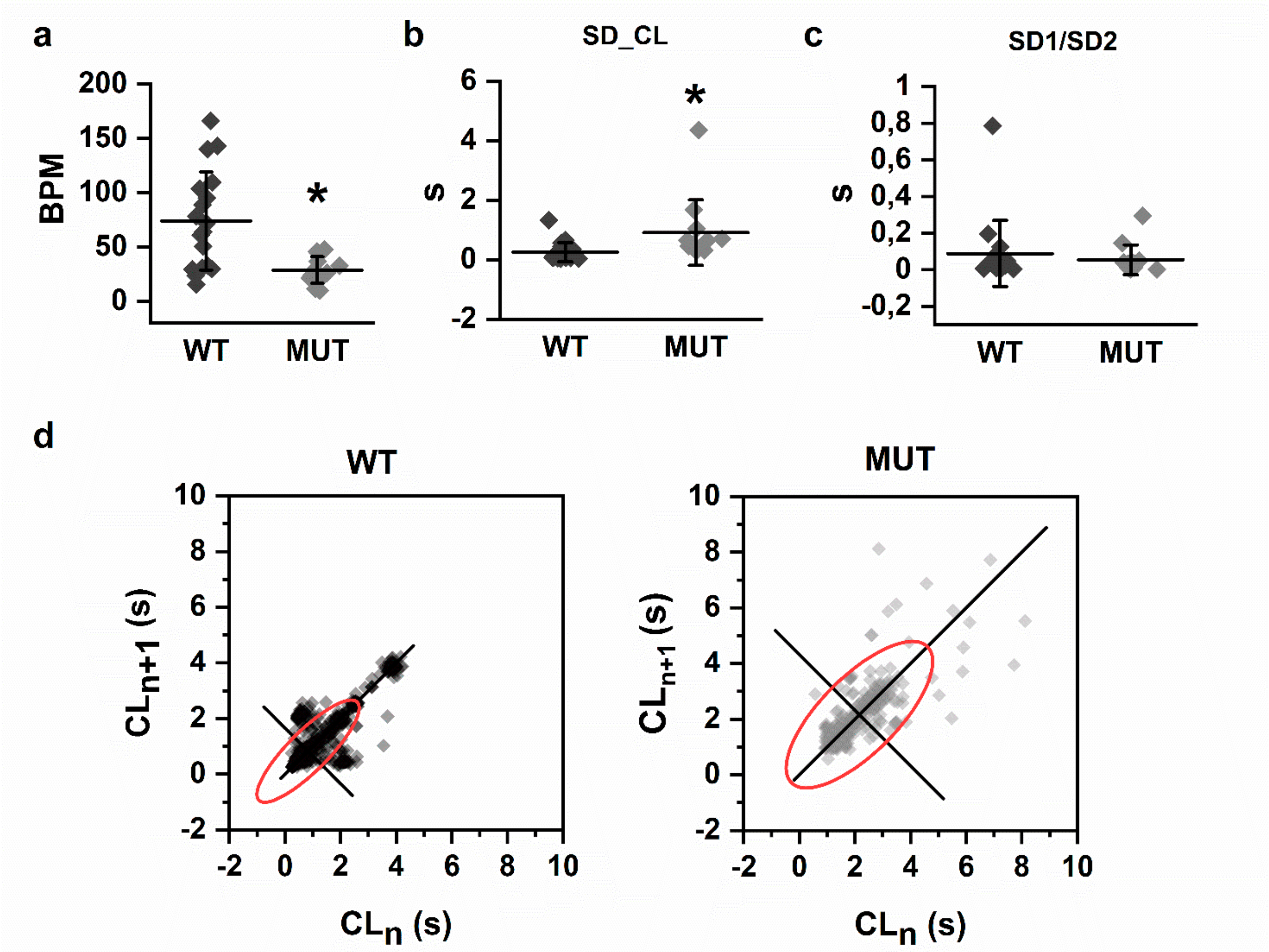

Figure S4. Beating parameters in WT and MUT. **(a)** Beating rate (BPM), **(b)** Standard deviation of CL (SD_CL); **(c)** Ratio between SD1 and SD2; **(d)** Representative Poincarè CL plots in WT (left) and MUT (right). Data are expressed as mean ± SD; WT n= 18; MUT n= 13; * $p<0.05$ vs WT.

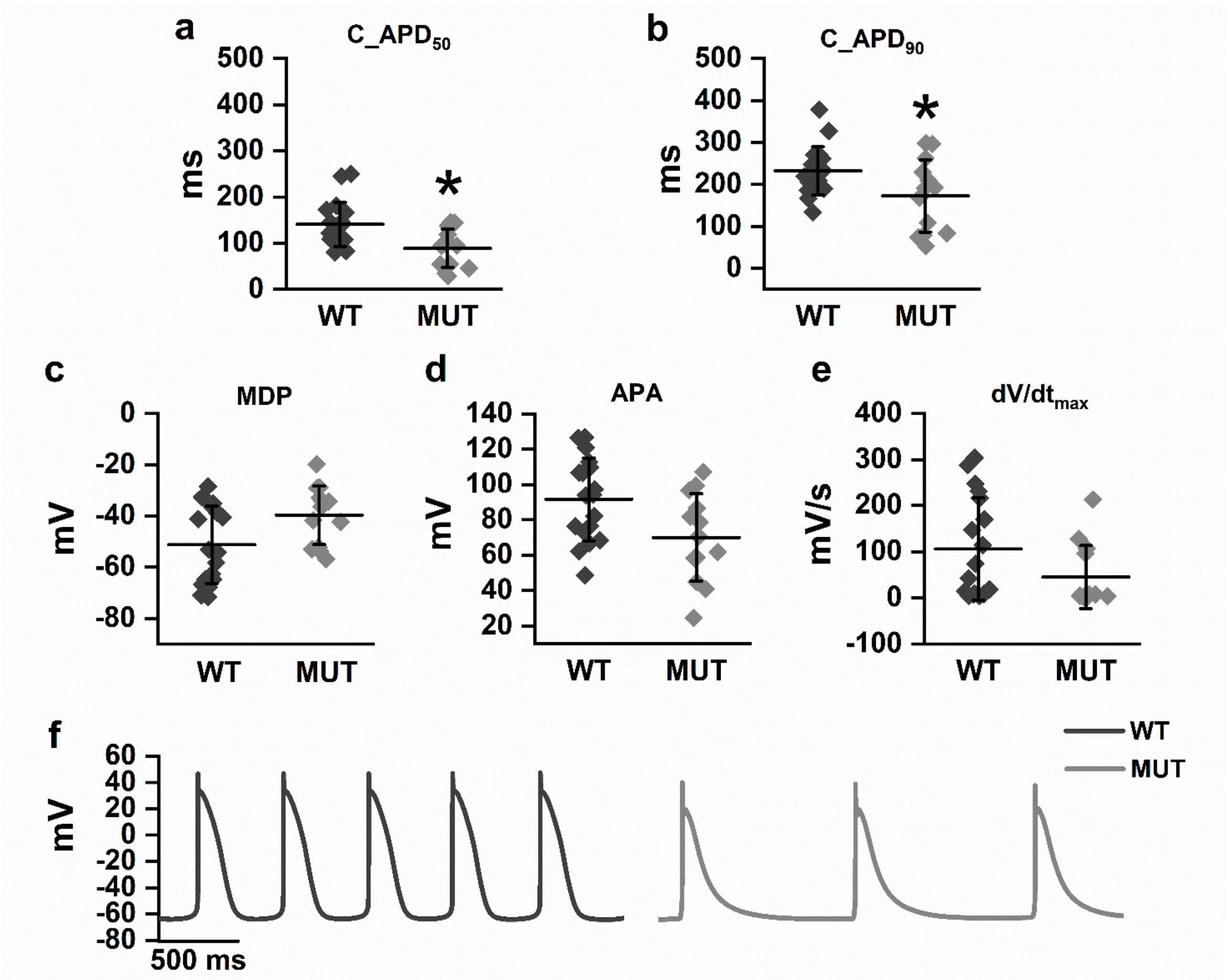

Figure S5. AP parameters during spontaneous beating in WT and MUT. **(a-b)** Rate-corrected APD at 50 and 90% repolarization (C_APD50, C_APD90); **(c)** maximal diastolic potential (MDP); **(d)** AP amplitude (APA); **(e)** Maximal upstroke velocity (dV/dt_{max}); **(f)** Representative AP recordings from WT (black) and MUT (grey). Data are expressed as mean ± SD; WT n= 18; MUT n= 13; * $p<0.05$ vs WT. See manuscript for discussion.

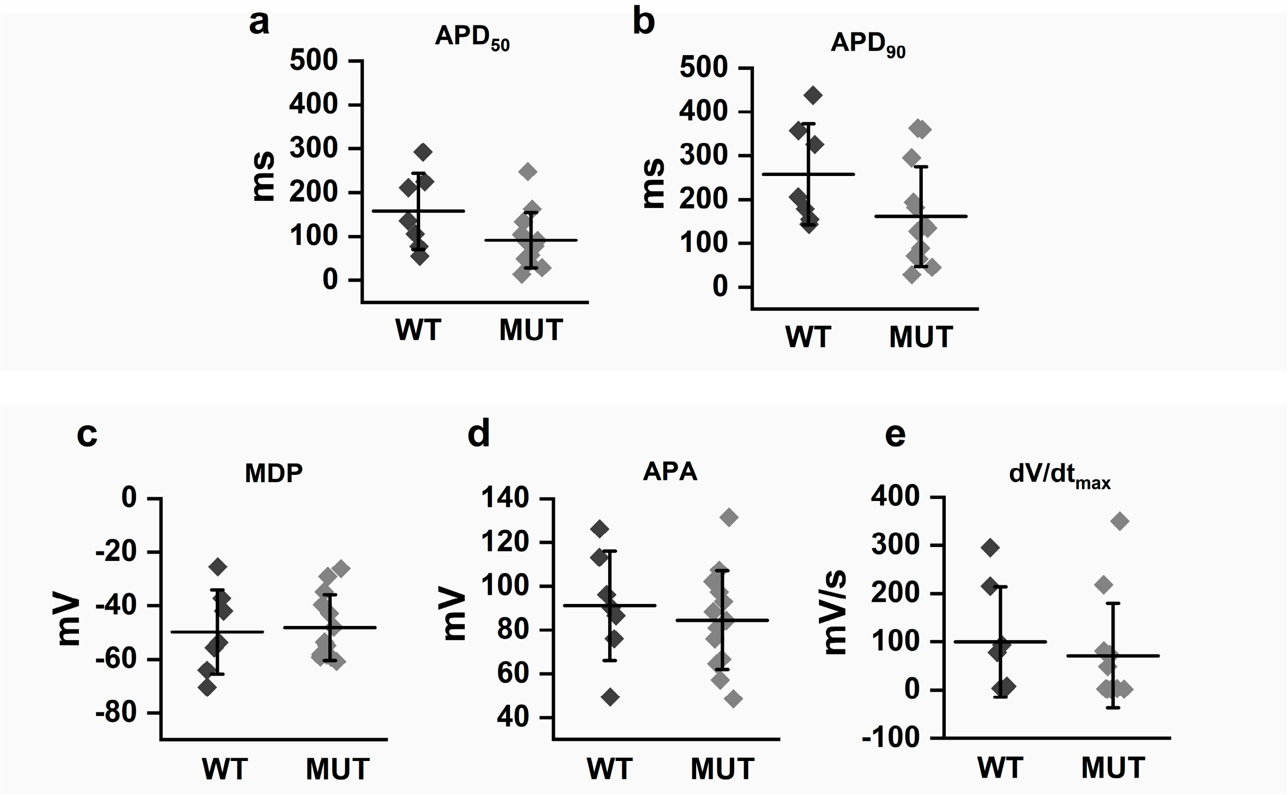

Figure S6. AP parameters during constant rate (1 Hz) pacing in WT and MUT. **(a-b)** APD at 50 and 90% repolarization (APD50, APD90); **(c)** Maximal diastolic potential (MDP); **(d)** AP amplitude (APA); **(e)** Maximal upstroke velocity (dV/dt_{max}). Data are expressed as mean ± SD; WT n= 7; MUT n= 13. See manuscript for discussion.

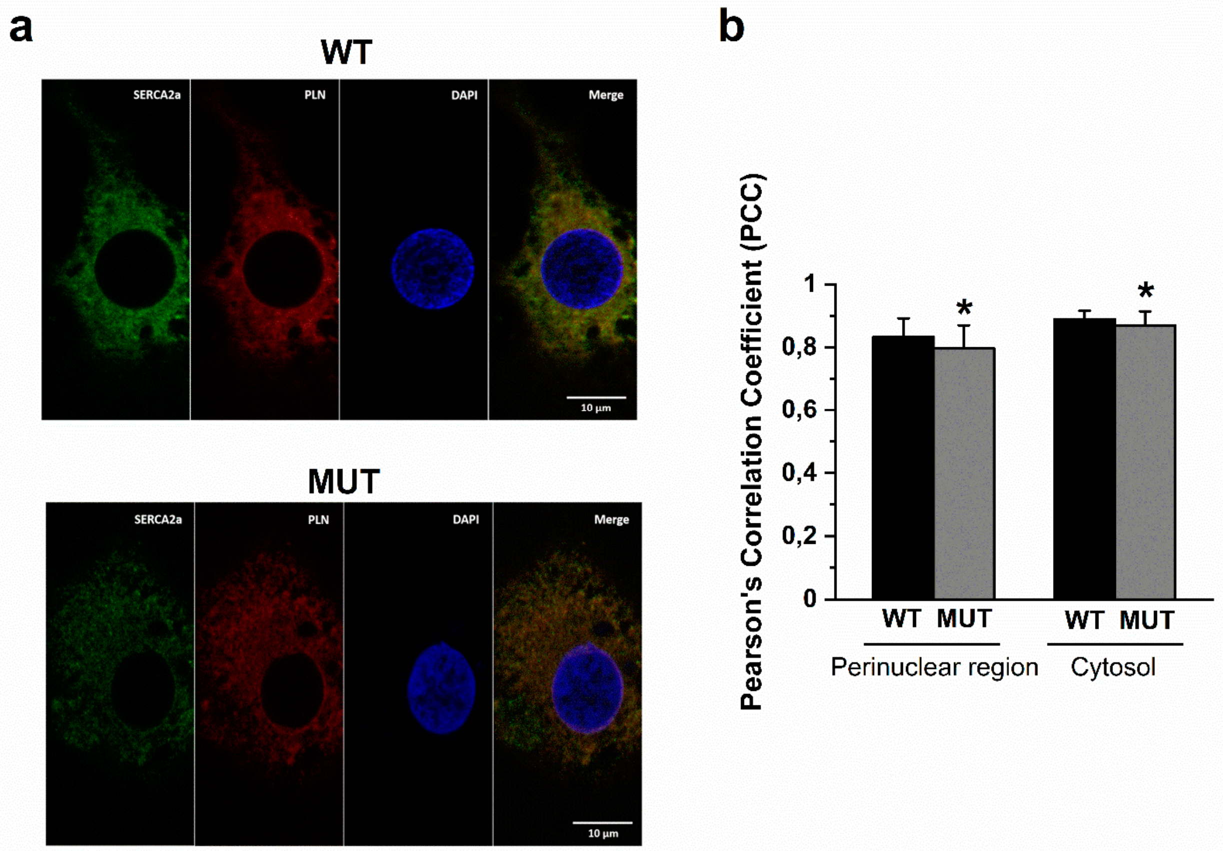

Figure S7. PLN and SERCA2a colocalization in single hiPSC-CM. **(a)** Representative immunolabeling of PLN, SERCA2a and their overlap in a WT (top) and MUT (bottom) hiPSC-CM. Green=SERCA2a, red=PLN, blue=DAPI. **(b)** PCC value for perinuclear and cytoplasmic PLN/SERCA2a colocalization in WT and MUT (n = 30). Data are expressed as mean ± SD; * = p<0.05 vs WT. See manuscript for discussion.

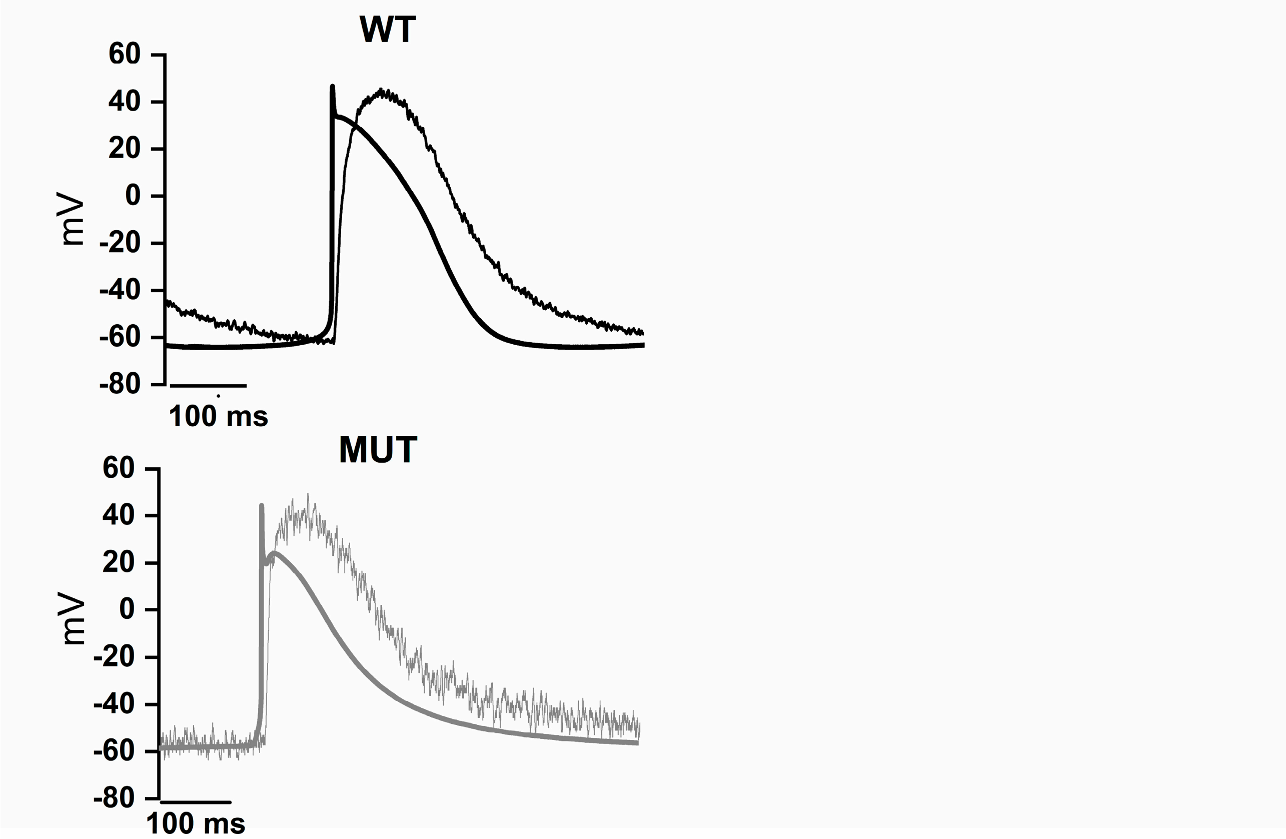

Figure S8. Temporal relationship between membrane potential and Ca^{2+} transients. Membrane potential and cytosolic Ca^{2+} were simultaneously recorded from spontaneously beating WT and MUT hiPSC-CMs. The two signals are superimposed (the Ca^{2+} signal, in arbitrary units, has been scaled to match action potential amplitude) to highlight their temporal relationship.
